# Supplementary material for: Timing of Measles, Mumps, and Rubella Vaccination: Secondary Outcomes from an Immunological Survey
Source: Vaccines (Basel). 2025 Apr 3;13(4):382. doi: 10.3390/vaccines13040382 (PMC12031130; doi:10.3390/vaccines13040382)
Supplement: Supplementary file 1 [file vaccines-13-00382-s001.zip › vaccines-3528699-supplementary.pdf]

Supplementary Material

Supplementary Table S1. Timing of vaccination with the first dose of the MMR vaccine. Immunological Survey 2018, Slovakia.

**Timing of MMR1**	Number	%
1st year	27	1.1%
13th -14th month	43	1.7%
15th month	386	15.1%
16th month	1196	46.7%
17th month	361	14.1%
18th month	194	7.6%
19th – 24th month	248	9.7%
3rd year	58	2.3%
Over 3rd year	47	1.8%
**Total**	**2560**	**100%**

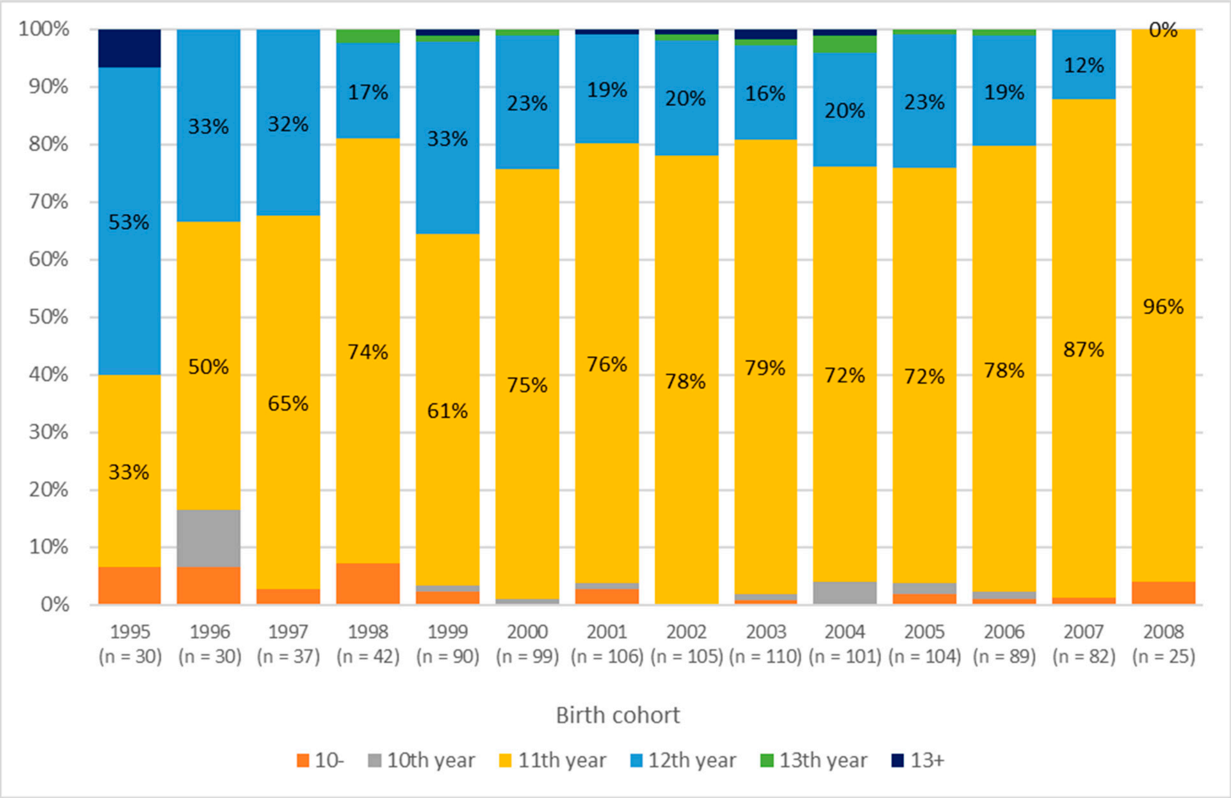

Supplementary Figure S1. Analysis of the timing of MMR2 vaccine, split by birth cohort. Immunological Survey 2018, Slovakia.
